# Alginate-Agarose Hydrogels Improve the In Vitro Differentiation of Human Dental Pulp Stem Cells in Chondrocytes. A Histological Study

**DOI:** 10.3390/biomedicines9070834

**Published:** 2021-07-17

**Authors:** María Oliver-Ferrándiz, Lara Milián, María Sancho-Tello, José Javier Martín de Llano, Fernando Gisbert Roca, Cristina Martínez-Ramos, Carmen Carda, Manuel Mata

**Affiliations:** 1 Department of Pathology, Faculty of Medicine and Odontology, University of Valencia, Avda. Blasco Ibáñez, 17, 46010 Valencia, Spain; oliverferrandizmaria@gmail.com (M.O.-F.); lara.milian@uv.es (L.M.); mardella@uv.es (J.J.M.d.L.); carmen.carda@uv.es (C.C.); manuel.mata@uv.es (M.M.); 2 Health Research Institute Foundation (INCLIVA), Menéndez y Pelayo St., 4, 46010 Valencia, Spain; 3 Center for Biomaterials and Tissue Engineering, Universitat Politècnica de València, Cno. de Vera, s/n, 46022 Valencia, Spain; nandogisbert@gmail.com; 4 Unit Predepartamental of Medicine, Jaime I University, Avda. Sos Baynat, s/n, 12071 Castellón de la Plana, Spain; cris_mr_1980@hotmail.com; 5 Center for Biomedical Research Network in Bioengineering, Biomaterials and Nanomedicine (CIBER-BBN), Melchor Fernández Almagro St., 3, 28029 Madrid, Spain; 6 Center for Biomedical Research Network in Respiratory Diseases (CIBER-ES), Melchor Fernández Almagro St., 3, 28029 Madrid, Spain

**Keywords:** cartilage regeneration, hDPSCs, MACI, alginate, agarose, chondrocyte, tissue engineering

## Abstract

Matrix-assisted autologous chondrocyte implantation (MACI) has shown promising results for cartilage repair, combining cultured chondrocytes and hydrogels, including alginate. The ability of chondrocytes for MACI is limited by different factors including donor site morbidity, dedifferentiation, limited lifespan or poor proliferation in vitro. Mesenchymal stem cells could represent an alternative for cartilage regeneration. In this study, we propose a MACI scaffold consisting of a mixed alginate-agarose hydrogel in combination with human dental pulp stem cells (hDPSCs), suitable for cartilage regeneration. Scaffolds were characterized according to their rheological properties, and their histomorphometric and molecular biology results. Agarose significantly improved the biomechanical behavior of the alginate scaffolds. Large scaffolds were manufactured, and a homogeneous distribution of cells was observed within them. Although primary chondrocytes showed a greater capacity for chondrogenic differentiation, hDPSCs cultured in the scaffolds formed large aggregates of cells, acquired a rounded morphology and expressed high amounts of type II collagen and aggrecan. Cells cultured in the scaffolds expressed not only chondral matrix-related genes, but also remodeling proteins and chondrocyte differentiation factors. The degree of differentiation of cells was proportional to the number and size of the cell aggregates that were formed in the hydrogels.

## 1. Introduction

Articular cartilage is a special form of connective tissue that, in its mature form, is characterized by poor regenerative capacity. This is because, on the one hand, it is an avascular tissue, and on the other hand it lacks a perichondrium and consequently a repository of stem cells with chondrogenic capacity [1]. In this way, the articular cartilage is subjected to numerous aggressions that lead to chronic inflammation and pain, causing irreversible degeneration in some cases. Degenerative processes are usually related to different pathologies that include trauma, neoplasia or osteoarthritis (OA), among others [2]. However, the incidence of articular cartilage injuries is higher than is believed, being an alteration typical of aging or sustained physical activity. In fact, 60% of young patients undergoing arthroscopic procedures are reported to have lesions in the hyaline articular cartilage [3].

Currently there are different therapeutic strategies for the treatment of chondral lesions, among which are bone marrow stimulation techniques (microperforations or Pridie drilling) or osteochondral transfer (autologous mosaicplasty and allogenic graft), among others [4]. Unfortunately, these therapies have shown low effectiveness in treating chondral injuries. In some cases, the gold standard treatment strategy is total joint arthroplasty, where the native cartilage and some of the underlying bone tissues are removed and replaced with metallic and polymeric components. Total joint arthroplasty successfully reduces pain, but causes long-term problems related to differences in stiffness of the implanted materials and native cartilage. Besides, arthroplasty is not indicated for young patients [5]. 

Matrix-assisted autologous chondrocyte implantation (MACI) has shown promising results for cartilage repair, through the combined use of cultured chondrocytes and hydrogels [6]. Concerning chondrocytes, they present different factors that limit the ability of MACI to treat large cartilage defects, including donor site morbidity, dedifferentiation and limited lifespan of chondrocytes or the low cell numbers obtained of cells obtained after harvesting, therefore, its use in patients with OA is excluded [7,8,9]. Adult mesenchymal stem cells (MSCs) do not have these disadvantages. Furthermore, their relative high availability and potential for multilineage differentiation, especially to chondrocytes, makes them a realistic alternative for cartilage regeneration [8]. Adult MSCs from different sources, including the placenta, umbilical cord, amniotic fluid, adipose tissue, synovium, or dental pulp, have been shown to be effective for cartilage regeneration in different experimental models [10]. hDPSCs are self-renewing MSCs that reside within the perivascular niche of the dental pulp [11,12,13], are believed to originate from the cranial neural crest, and express both mesenchymal and neural stem cell markers [14]. hDPSCs are easily obtained from extracted third molars and, under specific conditions, can differentiate in vitro into a variety of cell types including neurons, odontoblasts, osteoblasts, adipocytes, and chondrocytes [15,16,17]. 

Sodium alginate is one of the most widely used hydrogels in MACI. It gels in the presence of divalent cations such as Ca^2+^, generating a hydrogel similar to an extracellular matrix. Not only the chondrogenic properties of alginate, but also the chemical characteristics, including easy handling and desirable properties for use in technologies such as layer-by-layer, make alginate a good option to be used in MACI [18,19,20]. Moreover, alginate gel combined with hDPSCs has been effectively used in the regeneration of articular cartilage in vivo as we previously reported [21]. However, poor cell adhesion to alginate, low consistency and in vitro and in vivo degradation of alginate [22] are some of the disadvantages of its use in MACI. 

Agarose is a polysaccharide extracted from red seaweeds. It is a linear polymer made by repeating units of agarobiose, a disaccharide formed by D-galactose and 3,6-anhydro-L-galactopyranose. Agarose has been widely used in biomedical applications due to its controlled self-gelling properties, water-solubility, adjustable mechanical properties, and non-immunogenic properties. Agarose, based on its stiffness and functional groups, can support cellular adhesion, proliferation, and activity. Agarose has adjustable water adsorption capacity, providing cells with a suitable microenvironment for cellular activity [23]. Agarose-based hydrogels have been used in the investigation of the reaction that causes mechanical loading in chondrocytes and mesenchymal stem cells, showing better biomechanical and chondrogenic induction capabilities than sodium alginate [24,25,26].

Autologous chondrocytes cultured in alginate-agarose hydrogels have been tested in 17 patients with large cartilage lesions, and their efficacy has been proven two years after implantation [27]. Nevertheless, the authors do not develop or cite any preclinical study. On the other hand, to our knowledge, the chondrogenic potential of alginate-agarose hydrogels in combination with hDPSCs has not been studied.

Therefore, the aim of the present study was to manufacture a mixed alginate-agarose hydrogel in combination with hDPSCs, suitable for cartilage regeneration. The manufactured scaffolds were characterized considering their rheological, histomorphometric and molecular biology properties. In this work we present some novel aspects in the use of combined alginate-agarose hydrogels in relation to different aspects such as: (i) the use of hDPSCs, in combination with mixed alginate agarose hydrogels, (ii) the comparison of the chondrogenic potential of hDPSCs with respect to primary human chondrocytes, (iii) a detailed histological study on the morphology and the synthesis of proteins that make up the chondral matrix, such as type II collagen or aggrecan, and (iv) a morphometric characterization relative to the number and size of cell aggregates characteristic of the chondral differentiation process. The results presented here support the use of alginate-agarose hydrogels in combination with hDPSCs for MACI.

## 2. Materials and Methods

### 2.1. Hydrogel Preparation and Cell Encapsulation 

Stock solutions of 6 and 3% sodium alginate were prepared as previously described [28]. Briefly, sodium alginate (alginic acid sodium salt from brown algae, Sigma-Aldrich, Madrid, Spain) was dissolved with continuous stirring in sterile solution (40 mM HEPES and 300 mM NaCl) prewarmed to 65 °C. Once the alginate was dissolved in the buffer and the solution cooled to RT, the pH was adjusted to 7.4 and the solution was autoclaved and stored at 4 °C until use.

In addition, 1 and 2% ultra-low melting type IX agarose stock solutions were prepared by dissolving the appropriate amount of agarose in sterile calcium-free phosphate-buffered saline (PBS, Sigma-Aldrich, Madrid, Spain), as previously reported [29]. 

To generate hydrogels, the agarose stock solution was heated at 37 °C on a hot plate until dissolved. The alginate stock solution was also pre-warmed at 37 °C. Then, both solutions were mixed to generate the different hydrogels used in this study. The 1.5% alginate-1% agarose solution was generated by mixing 1 mL of the 3% alginate stock solution and 1 mL of the 2% agarose stock solution. The 3% alginate-1% agarose solution was prepared by mixing 1 mL of the 6% alginate stock solution and 1 mL of 2% agarose stock solution. To generate agarose gels containing no alginate, the agarose stock solutions were diluted with sterile ultrapure water. In the case of cell-containing hydrogels, the cultured chondrocytes or hDPSCs (see below) were trypsinized and the isolated cells were resuspended in the alginate solution before mixing with the agarose stock solution at 37 °C.

To gel the hydrogels, a 10% volume of 102 mM CaCl_2_ in sterile water was first added to the alginate-agarose solutions to induce the crosslinking of the alginate. Then, the solutions were incubated at RT for 15 min to induce gelling of the agarose. Subsequently, the solutions were incubated at 4 °C for 15 min to stabilize the hydrogel. Each scaffold was manufactured using 250 µL of polymeric solution in 24-well culture plates. Once the procedure was finished, 250 mL of the appropriate culture medium was added on the top of the scaffold and incubated at 37 °C in a humidified atmosphere with 5% CO_2_.

### 2.2. Rheological and Mechanical Testing

A Discovery Hybrid Rheometer DHR (TA instruments, New Castle, DE, USA) was used for rheological and mechanical testing. In the test, the upper disk of the equipment rotated around its longitudinal axis and exerted a shear force on the material. The objective was to know the characteristics of the material when subjected to a shear stress, obtaining the value of the shear modulus (G) of the material. From this G value there are obtained the real part of the shear modulus (storage modulus, G’) and the imaginary part of the shear modulus (loss modulus, G’’) [30]. Circular scaffolds (25 mm diameter) were studied in a state of swelling in deionized water for 24 h. To manufacture them, we generated a chamber using two 12 cm side glass plates and two 0.7 cm spacers. We introduced 12 mL of polymer solution that was crosslinked inside said chamber. The pieces were then detached and a die was used to generate the discs. An oscillation test was performed at RT with torque amplitudes ranging from 10 to 100 µN*m taking 10 points per decade and applying a frequency of 1 Hz. 

Applying Equation (1) it is possible to estimate the tensile modulus (E) of the material through the shear modulus (G) and the Poisson’s ratio (ν). In addition, assuming that the studied hydrogels are homogeneous and isotropic elastomers at a macroscopic level, it can be assumed that ν ≈ 0.5, so the tensile modulus can be approximated as E ≅ 3∙G:

G = E/(2(1 + ν))(1)

### 2.3. Cell Isolation and Culture

Human primary chondrocytes and hDPSCs were isolated and characterized as previously described [21]. The study was conducted in accordance with the Declaration of Helsinki and applicable local regulatory requirements and laws. All procedures were approved by the Ethics Committee of the University Clinical Hospital of Valencia (Spain, identification code 2016/27, approved in April 2019) and by the Ethics Committee of the University of Valencia (Spain, identification code H1548322921081, approved in February 2019) respectively. All donors signed the informed consent. 

Articular cartilage was obtained from the knee joints of patients undergoing arthroscopy. The tissue was washed with Dulbecco’s modified Eagle’s medium (DMEM; Thermo Fisher Scientific, Fife, WA, USA) supplemented with 100 U penicillin, 100 μg streptomycin (Biological Industries, Kibbutz Beit Haemek, Israel), and 0.4% fungizone (Gibco/Thermo Fisher Scientific, Madrid, Spain). Cartilage in supplemented DMEM was diced and digested with the following enzymes. First, the cartilage was incubated with 0.5 mg/mL hyaluronidase (Sigma-Aldrich, St. Louis, MO, USA) in a shaking water bath at 37 °C for 30 min. The hyaluronidase solution was removed and 1 mg/mL of pronase (VWR International, Barcelona, Spain) was added. After incubation in a shaking water bath at 37 °C for 60 min, the cartilage pieces were washed with the supplemented DMEM. The medium was then removed, and 0.5 mg/mL collagenase-IA (Sigma-Aldrich, Madrid, Spain) was added and digestion was continued overnight in a shaking water bath at 37 °C. The resulting cell suspension was filtered through a 70-µm pore nylon filter (BD Biosciences, San Jose, CA, USA) to remove tissue debris. The cells were centrifuged, and the cell pellet was washed with DMEM supplemented with 10% fetal bovine serum (FBS; Thermo Fisher Scientific, Madrid, Spain). Finally, the isolated cells were cultured at 37 °C in a 5% CO_2_ and 95% air humidified atmosphere in proliferation medium containing DMEM supplemented with 10% heat-inactivated FBS, 50 µg/mL ascorbic acid (Sigma-Aldrich, Madrid, Spain), 1% non-essential amino acids, 1% pyruvate, 100 U penicillin, 100 μg streptomycin, and 1% fungizone. 

hDPSCs were isolated from the dental pulp of human third molars. The dental pulp was gently removed under sterile conditions using cow horn forceps with a small excavator and transferred to a tube containing Hank’s solution (Gibco, Madrid, Spain). The specimens were then divided into small pieces using a scalpel blade, and dispase digested for 2 h at 37 °C in 5% CO_2_ and 95% air. The supernatant medium was removed, and 0.1% type IV collagenase (Sigma-Aldrich) was added for 15 min, followed by centrifugation at 1500 rpm for 10 min. The supernatant was removed, and the cells were seeded in 25 cm^2^ flasks and expanded in proliferation medium (α-MEM supplemented with 10% FBS, 1 mM L-glutamine, 1% antibiotics and fungizone).

Chondral differentiation was induced by incubating expanded chondrocytes or hDPSCs in differentiation medium (DMEM containing 1% insulin-transferrin-sodium selenite medium supplement (BD Biosciences, Madrid, Spain), 50 µg/mL ascorbic acid, 10 ng/mL TGF-β1, and 1% heat-inactivated FBS).

### 2.4. Flow Cytometry Characterization of hDPSCs 

The hDPSCs were characterized using a FACSCalibur equipped with a 488-nm argon laser and a 635-nm red diode laser (Becton Dickinson, Madrid, Spain) as previously described [21]. The experimental data were analyzed using CellQuest software (Becton Dickinson, Madrid, Spain). To exclude cellular debris, samples were sorted based on light-scattering properties in the side-scattered and forward-scattered light modes, and 10,000 events per sample were collected within this gate (R1), using the medium setting for the sample flow rate. The following markers were evaluated: CD29 (Alexa Fluor® 488), CD31 (PE/Cy7), CD44 (PE/Cy5), CD45 (Brilliant Violet 510), CD105 (APC), CD146 (PE) and STRO-1 (APC). 

### 2.5. Immunofluorescence Staining of Type I Collagen, Type II Collagen, and Aggrecan

The expression of type I collagen (COLI), type II collagen (COLII), and aggrecan (ACAN) was determined in cell culture using specific antibodies (Sigma-Aldrich, Madrid, Spain). Cells were cultured with proliferation or differentiation culture media for up to 6 weeks as described above. Then, cells were fixed with 4% paraformaldehyde in PBS pH 7.4 for 10 min. Once washed with PBS, the cells were permeabilized with 0.1% Triton X-100 in PBS for 5 min, and after three washes, they were incubated for 30 min with blocking solution (1% bovine serum albumin [BSA] and 1.1% Tween-20 in PBS). The cells were then incubated with the appropriate primary antibody (diluted in antibody diluent solution at 1:100 for COLI and ACAN and 1:500 for COLII antibody) overnight at 4 °C. After three washes, cells were incubated with the secondary anti-mouse (COLI and COLII) or anti-rabbit (ACAN) FITC-conjugated antibody (Sigma-Aldrich, Madrid, Spain) diluted 1:200. After the final washes, the nuclei were stained with 4’,6-diamidino-2-phenylindole (DAPI) and the samples were analyzed with a Leica DM2500 fluorescence microscope (Leica, Wetzlar, Germany).

### 2.6. Fluorescence Staining of F-Actin

F-actin was evaluated using rhodamine-conjugated phalloidin (Molecular Probes, Thermo Fisher Scientific, Madrid, Spain). Cells were grown in the appropriate culture medium to subconfluence, washed with PBS pH 7.4 and fixed in a 3.7% solution of formaldehyde in PBS for 10 min at RT. The cells were permeabilized with 0.1% Triton X-100 in PBS for 3–5 min. Then, samples were pre-incubated with PBS containing 1% BSA for 20–30 min to reduce non-specific background staining. Each sample was then stained for 20 min with 5 µL phalloidin methanol stock solution diluted in 200 µL PBS. Finally, after washing the samples several times with PBS, the nuclei were stained with DAPI and analyzed with a DM2500 fluorescence microscope (Leica, Madrid, Spain).

### 2.7. Alginate-Agarose Scaffolds Staining with Hematoxylin-Eosin and Sirius Red 

Scaffolds were fixed for 4 h in a 4% paraformaldehyde solution at 4 °C and washed overnight at RT in a solution containing 100 mM sodium cacodylate and 50 mM BaCl_2_ at pH 7.4. The scaffolds were then dehydrated following standard procedures and embedded in a solution containing paraffin wax (Sigma-Aldrich, Madrid, Spain) and absolute ethanol (1:1), overnight at 37 °C. Next, the samples were incubated for 1 h in 100% paraffin wax and finally the blocks were made at RT in a dry atmosphere using silica gel as desiccant (Sigma-Aldrich, Madrid, Spain). Five-µm slices were obtained, deparaffinized, rehydrated and stained with hematoxylin-eosin following standard procedures. For Sirius red staining, slices were incubated with a Sirius red solution (1% Sirius Red F3BA in saturated picric acid aqueous solution) for 45 min at RT. The samples were then dehydrated and mounted with Entellan (Merck, Madrid, Spain). A Leica DM4000 microscope and digital camera DFC (Leica, Madrid, Spain) were used to analyze the samples.

### 2.8. Ki67 Expression Analysis

Ki67 expression was detected by immunohistochemistry and measured by real-time RT-PCR, as previously reported [31]. Immunohistochemical analysis was carried out on 5-µm slices of chondrocyte or hDPSC, cultured for 6 weeks with proliferation or differentiation media, obtained as detailed in Section 2.7. Slices were deparaffinized and rehydrated using a series of graded ethanol, rinsed with distilled water, and treated with 0.3% H_2_O_2_ to block endogenous peroxidase. Then, nonspecific binding was blocked by washing with Tween 20 buffer (Fischer Scientific, Madrid, Spain). Antigens were retrieved by boiling in a pressure cooker for 3 min in high pH Envision™ FLEX Target Retrieval Solution (Dako, Barcelona, Spain). Samples were incubated overnight with the primary antibody (ready-to-use monoclonal mouse anti-human ki67 antigen, IR46, DAKO, Madrid Spain) at RT for 30 min. After washing with PBS, the secondary antibody (goat anti-mouse IgG-HRP, 1:200 dilution) was incubated at RT for 2 h and then developed using the chromogen 3,3′-diaminobenzidine (Dako, Madrid, Spain) according to manufacturer’s instructions, which resulted in brown staining in the immunoreactive structures. Finally, the sections were counterstained with Mayer’s hematoxylin (Sigma-Aldrich, Madrid, Spain). Real-time RT-PCR of chondrocytes or hDPSC, cultured for 1 to 6 weeks with proliferation or differentiation media, was carried out as described in Section 2.10 of this manuscript, using previously described primers, (forward: TCCTTTGGTGGGCACCTAAGACCTG and reverse: TGATGGTTGAGGTCGTTCCTTGATGT) [31].

### 2.9. Morphometric Analysis of Cell Aggregates

Morphometric analysis of chondrocytes and hDPSCs cultured in alginate-agarose hydrogels was performed using the Image-Pro plus 7 software (Media Cybernetics, Rockville, MD, USA). Cells were cultured for up to 6 weeks and stained with rhodamine-conjugated phalloidin as described above. Serial confocal images were captured, and the following parameters were evaluated: number of aggregates (groups larger than 4 cells), number of cell nuclei in the aggregate (according to DAPI staining), maximum diameter of the aggregate, number of cells that do not form aggregates and total number of cells. At least ten different fields from each sample were evaluated double-blind.

### 2.10. Relative Gene Expression Analysis

Total RNA was extracted from 2D cultures using Trizol reagent (Thermo Fischer Scientific Inc., Waltham, MA, USA) according to the manufacturer’s instructions. RNA concentration was determined by spectrophotometry using a Nanodrop 2000 spectrophotometer (Fischer Scientific, Madrid, Spain). Only extracts with a ratio 260/280 > 1.8 were used. RNA integrity (RI) was evaluated by capillary electrophoresis using a Bioanalyzer (Agilent Technologies, Santa Clara, CA, USA). For the determination of gene expression levels, only extracts with a RI number (RIN) of ~10 was used. In the case of cells cultured in alginate-agarose scaffolds, the standard protocol with Trizol failed, so a modification of the one developed by Ogura et al. [32] was used. Briefly, scaffolds with cells were incubated in 500 µL of Trizol reagent for 5 min at RT and 250 µL of 100% ethanol were added. Samples were vortexed and incubated for 5 min at RT. The suspension was transferred to a QIA shredder column included in the RNeasy Plant Mini kit (Quiagen Iberia SL, Madrid, Spain) and centrifuged for 2 min at 12,000 rpm. The eluted fraction was transferred to a microcentrifuge tube, mixed with 500 µL of absolute ethanol, transferred to the second column of the RNeasy Plant kit and centrifuged for 15 s at 12,000 rpm. The column was washed with RW1 and RPE buffer according to the manufacturer’s instructions. Finally, the RNA was eluted with 30 µL of RNase-free water and quantitated as described above. 

Random hexamers were used to synthesize complementary DNA (cDNA) using TaqMan RT reagents (Applied Biosystems, Foster City, CA, USA) following the manufacturer’s instructions. Gene expression levels were assayed by reverse transcriptase polymerase chain reaction (RT-PCR) using on Demand Assays (Applied Biosystems, Madrid, Spain). The details regarding the genes and the number of assays are summarized in Table 1. The reactions were carried out in a 7900HT real-time Thermocycler (Applied Biosystems, Madrid, Spain). The comparative ΔΔCt method with glyceraldehyde 3-phosphate dehydrogenase (GAPDH) was used as an endogenous control to calculate relative gene expression levels [33], which expresses the fold-variation in the experimental group with respect to the control group.

## 3. Results

### 3.1. Mechanical Testing of Alginate-Agarose Hydrogels

The mechanical characterization of the manufactured hydrogels was first tested. The following hydrogels were studied as described in the methods section: 3% and 1.5% alginate, 0.5% and 1% agarose, 1.5% alginate–1% agarose, and 3% alginate–1% agarose. When the hydrogels were prepared, the storage modulus (G’) and loss modulus (G’’) were calculated at RT applying torque amplitudes ranging from 10 to 100 µN*m. The results obtained for a torque value of 50 µN*m are summarized in Table 2 including the estimation of the elastic modulus (E). No measurements could be made on the 3% and 1.5% alginate hydrogels because the resulting gels were too soft.

**Figure 1:**
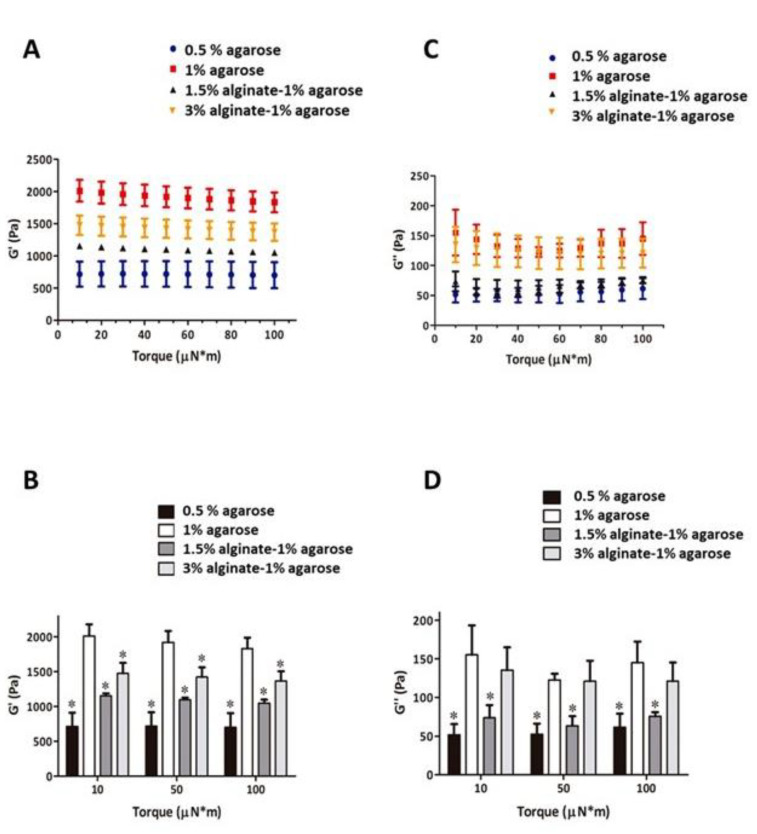
Mechanical test of hydrogels. Hydrogels of 0.5% agarose, 1% agarose, 1.5% alginate-1% agarose, and 3% alginate-1% agarose were manufactured and the storage modulus (G’, **A**,**B**) and loss modulus (G’’, **C**,**D**) were calculated at RT, using a torque of 50 µN*m. The results represented are means ± SD of *n* = 10 different experiments.

As shown in Figure 1A,B, there was an increase in G in the 1% agarose samples compared to the 0.5% agarose samples, almost doubling the G value for all pairs of oscillation values. This implies that the addition of more agarose allowed the material to store a greater amount of energy without undergoing permanent deformation, which indicates that the material presented greater rigidity. It is also interesting to note that a slight decrease in G’ values was observed as the oscillation torque increased, especially for 1% agarose. This non-linear behavior of the material shows that, for high values of the oscillation torque, it presents a loss of its resistance to the applied force compared to the low values. This phenomenon, known as deformation softening or strain softening, could be due to the accumulation of defects as the amplitude of deformation increases, something that was not observed as clearly in the case of 0.5% agarose.

With respect to the samples containing alginate (1.5% alginate–1% agarose and 3% alginate–1% agarose), a decrease in G’ was observed, indicating that the materials were less rigid than 1% agarose. It seems that alginate adversely affects the strength of the material, making it less resistant.

Regarding the values obtained for the loss modulus (G’’), they were lower than those previously obtained for G, as shown in Figure 1C,D. This suggest that most of the material was able to recover its internal structure and its original shape even though part of the material resulted irreversible deformed. Agarose markedly increased the G modulus, while alginate-containing hydrogels showed intermediate values to those of the 0.5% and 1% agarose hydrogels. In general, a non-linear behavior is observed for G’’ that may also be due to the strain-softening phenomenon. According to the data obtained, we selected the combination of 3% alginate and 1% agarose was selected for subsequent experiments.

### 3.2. Cell Characterization and Chondrocyte Differentiation in 2D Culture

hDPSCs were isolated as described in the methods and cultured with proliferation medium. Once expanded, the cells were characterized by flow cytometry (charts not shown). Cultured cells were positive for CD29 (89.42 ± 10.69% of cells), for CD146 (82.54 ± 12.98), for CD105 (82.65 ± 19.36) and for CD44 (92.36 ± 6.39) and were negative for CD45 (3.98 ± 2.79) and for CD31 (1.22 ± 1.58). Regarding STRO1, 45.37 ± 48.64% of the cells analyzed were positive. Concerning primary chondrocytes, we assumed that the cultured cells were of the chondral lineage, since they were obtained from cartilage tissue. 

**Figure 2:**
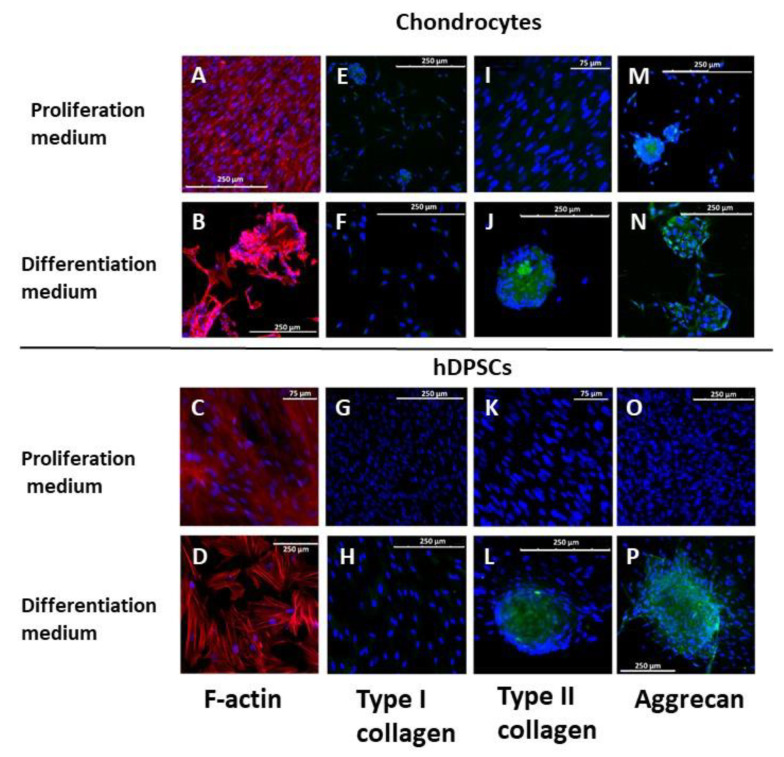
2-D cultures of human primary chondrocytes (upper panels) and hDPSCs (lower panels). Cells were cultured with proliferation or differentiation medium for up to 4 weeks. F-actin organization was evaluated by fluorescence microscopy using rhodamine-phalloidine (**A**–**D**). Type I (**E**–**H**) and II (**I**–**L**) collagen, as well as aggrecan (**M**–**P**) were evaluated by immunofluorescence. Nuclei were staining with DAPI. The images are representative of *n* = 5 different experiments.

The cultured cells were then tested for their chondrocyte differentiation ability. The cells were cultured in 2D with proliferation or differentiation culture media for 4 weeks, and the cytoskeletal organization of the F-actin filaments was evaluated by fluorescence microscopy using rhodamine-phalloidin. The results obtained are shown in Figure 2A–D. The increase in the number of chondrocytes cultured with proliferation medium was evident (Figure 2A) and displayed a fibroblast-like morphology, with the labeled F-actin filaments arranged across the cytoplasm. Chondrocytes cultured with differentiation medium tended to form aggregates which were often large in size. Cells, especially those that formed aggregates, displayed a more rounded morphology and changes related to F-action filaments, which were scarcer in the center of the cell, thus exhibiting a preferentially peripheral distribution. hDPSCs cultured with proliferation medium showed a characteristic mesenchymal phenotype, as shown in the Figure 2C. A lower number of cells was observed in cells cultured with differentiation medium. These cells showed a more spread phenotype compared to cells cultured with proliferation medium (Figure 2D). Cell aggregates appeared in some of the cultures studied, but their quantity was always much lower than that observed in primary chondrocyte cultures.

The expression of type I and II collagen, as well as aggrecan, was studied by immunofluorescence. Neither chondrocytes nor hDPSCs significantly expressed type I collagen, under any of the experimental condition tested (Figure 2E–H). Chondrocytes cultures with differentiation medium expressed type II collagen. The expression of type II collagen was higher in cells forming aggregates than in individual cells, not all of which expressed type II collagen (Figure 2J). In the case of hDPSCs cultures, only cells that formed aggregates expressed that protein (Figure 2L). Aggrecan was expressed in chondrocytes cultured in both culture media (Figure 2M–N). In hDPSCs cultures, its expression was limited to those cells that formed the scarce aggregates, which appeared anecdotally in cells cultured with differentiation medium (Figure 2P). 

**Figure 3:**
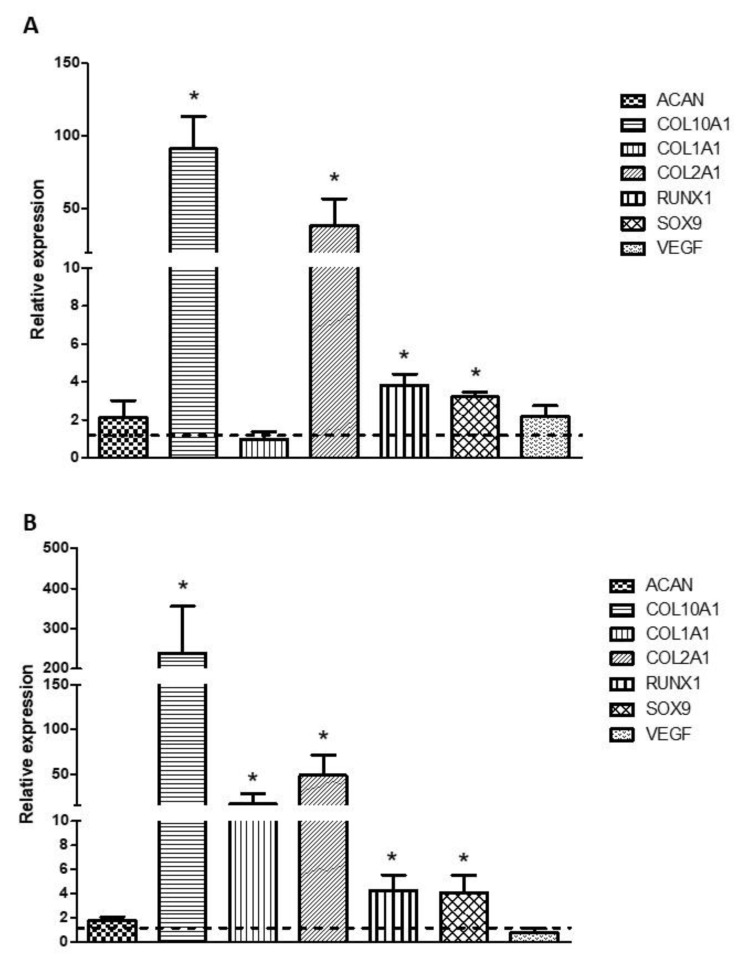
Chondrocyte-related gene expression levels. Human primary chondrocytes (**A**) or hDPSCs (**B**) were cultured with proliferation or differentiation medium for 4 weeks. The relative levels of gene expression of ACAN, COL10A1, COL1A1, COL2A1, ACAN, RUNX1, SOX9 and VEGF were evaluated by real-time RT-PCR. GAPDH was used as house-keeping gene. The fold change (2-∆∆Ct) was calculated using the relative expression of cells cultured with proliferation medium as a control group (indicated by the dotted line). Mean ± SD of three different experiments is represented. * *p* < 0.05 compared to control group.

The relative expression of chondrocyte-related genes was determined by real-time RT-PCR. The results obtained are shown in Figure 3. Gene expression of COL2A1 and COL10 was significantly induced in the chondrocytes cultured with differentiation medium, compared to those cultured with proliferation medium. ACAN expression showed a slight increase that was not statistically significant. The expression of RUNX1 and SOX9 was also significantly induced in chondrocytes cultured with differentiation medium. The rest of the genes analyzed (COL1A1 and VEGF) did not show significant differences (Figure 3A). Regarding hDPSC, all the genes analyzed but ACAN and VEGF showed significant increases in their expression in cells cultured with differentiation medium compared to those cultured with proliferation medium. 

### 3.3. Cell Encapsulation in Alginate-Agarose Scaffolds. Cell Organization and Synthesis of Collagen

**Figure 4:**
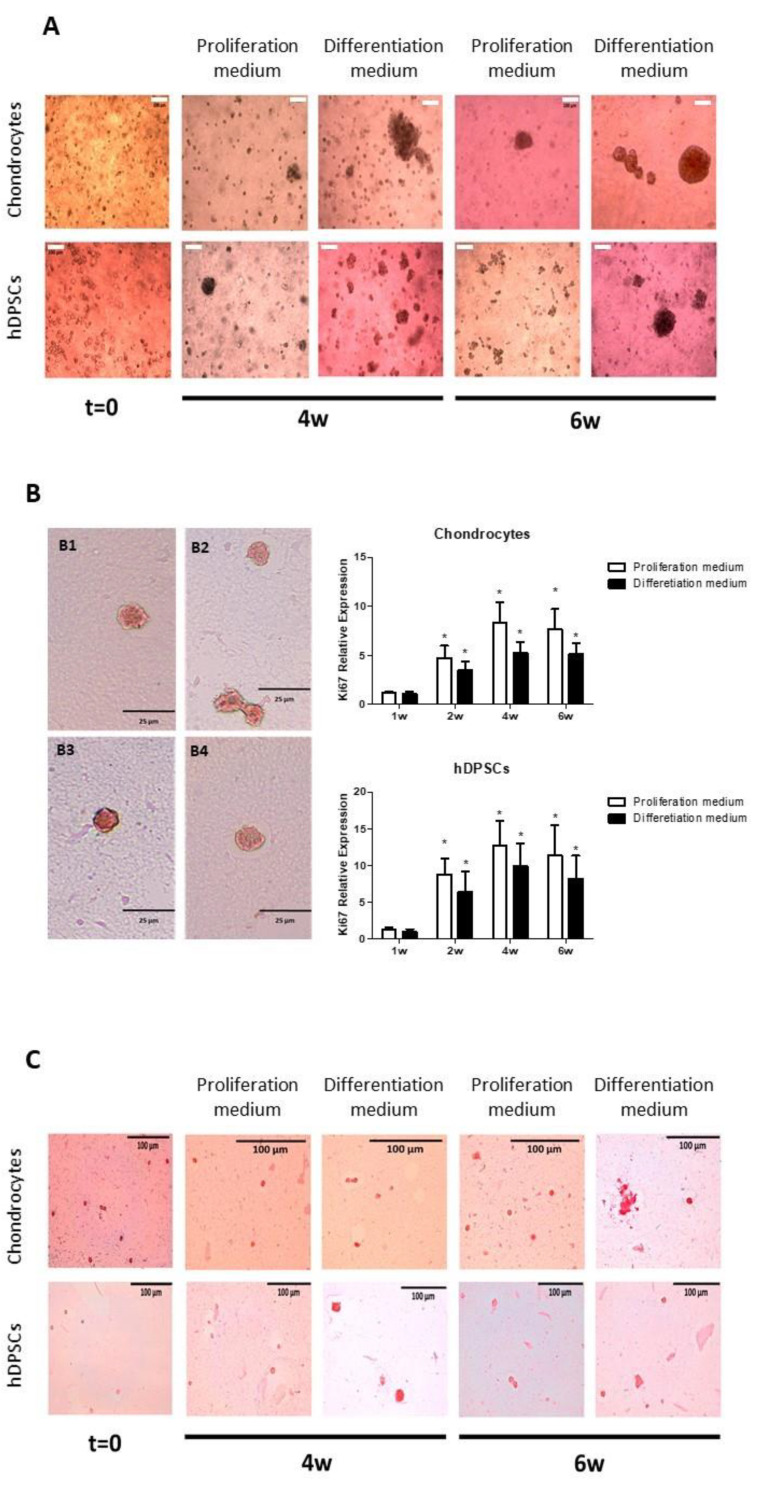
Encapsulation of cultured cells in alginate-agarose hydrogels. Human primary chondrocytes or hDPSCs were cultured in hydrogels containing 3% alginate and 1% agarose with proliferation or differentiation medium, for up to 6 weeks at a density of 2 × 10^6^ cells/mL. (**A**) Cell distribution was evaluated by phase contrast microscopy. (**B**) Cell proliferation was estimated by ki67 expression detected by immunohistochemistry (panels B1-4) and determined by real-time RT-PCR. Ki67-positive chondrocytes are shown in subpanel B1 (in proliferation medium), B2 (in differentiation medium), and ki67-positive hDPSC in B3 (in proliferation medium) and B4 (in differentiation medium), all cultured for 6 weeks. Graphs represent the expression of the Ki67 gene; data represent means ± SD of *n* = 3, * *p* < 0.05 compared to cells cultured for 1 week. (**C**) Collagen synthesis was studied by Sirius red staining. Representative microscopic images from three independent experiments are shown. Scale bars equal to100 µm (**A**,**C**) or 25 µm (**B**).

Human primary chondrocytes and hDPSCs were encapsulated in scaffolds of 3% alginate-1% agarose. To encapsulate cells, they were suspended in a 6% alginate solution at a density of 2 × 10^6^ cells/mL. The same volume of a 2% agarose solution was added, and a gel was formed as described in the Methods section. The cells were cultured with proliferation or differentiation culture media for up to 6 weeks. The progress of the cell culture was monitored under phase contrast microscopy. Representative images of scaffolds containing cells are shown in Figure 4A. An increased in both cell types was observed and the clustering of cells forming spherical aggregates was evident. 

The cellular proliferation of embedded cells was estimated by immunohistochemical detection of ki67 and measured by real-time RT-PCR in human chondrocytes and hDPSCs encapsulated in scaffolds and cultured with proliferation or differentiation medium for 1, 2, 4 and 6 weeks. The results obtained are summarized in Figure 4B. At week 6 of culture, approximately 60% of the cells were found to be ki67-positive. Representative images of ki67-positive chondrocytes are shown in subpanel B1 (in proliferation medium), B2 (in differentiation medium), and hDPSC in B3 (in proliferation medium) and B4 (in differentiation medium). In order to compare the expression of ki67 in the different experimental groups included in this work, we carried out a real-time RT-PCR study. Other authors have shown that this technique is as reliable as immunohistochemistry [32]. The results obtained indicate that, in the two cell types studied, proliferation increased significantly after two weeks of culture. This increase reached a maximum at 4 weeks and stabilized until week 6. Although a lower expression of ki67 was observed in the experimental groups cultured with differentiation medium instead of proliferation medium, this difference did not reach statistical significance. 

Sirius red staining was performed to investigate the ability of cells to produce collagen. The cells were encapsulated and incubated as described above. The results obtained are represented in Figure 4C. In the case of chondrocytes, all cells exhibited a red staining, even though the positive staining was more evident in the aggregates than in the isolated cells, as was also more evident in chondrocytes cultured with differentiation medium compared to those cultured with proliferation medium. Regarding hDPSCs, only the aggregates, which were markedly more abundant in the samples cultured with differentiation medium, were positive.

**Figure 5:**
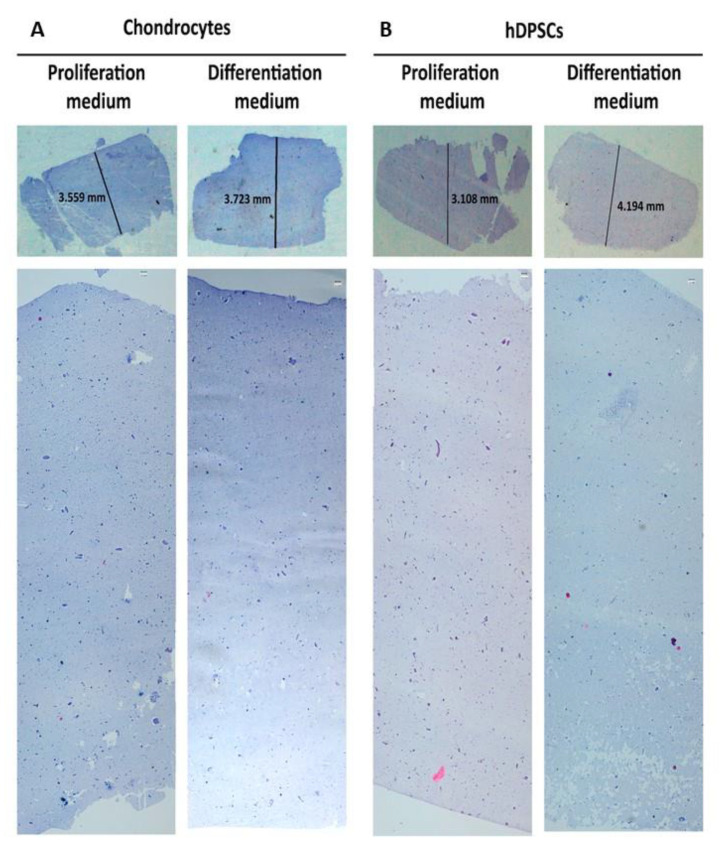
Distribution of cells along the manufactured hydrogels. Human primary chondrocytes (**A**) or hDPSCs (**B**) were cultured in hydrogels containing 3% alginate and 1% agarose, with proliferation or differentiation medium for up to 6 weeks at a density of 2 × 10^6^ cells/mL. Scaffolds were fixed, paraffin embedded and stained with hematoxylin eosin. Panoramic images are shown where the cell distribution is observed along the scaffolds. Representative results from three different experiments are shown. Numbers indicate the height. Scale bars = 100 µm.

Cell distribution inside the scaffold was also investigated using standard hematoxylin eosin staining. The results obtained are shown in Figure 5. In all the experimental conditions evaluated, a homogeneous distribution of cells was observed inside the scaffolds. 

### 3.4. Chondral Differentiation of Chondrocytes Cultured in Alginate-Agarose Scaffolds 

**Figure 6:**
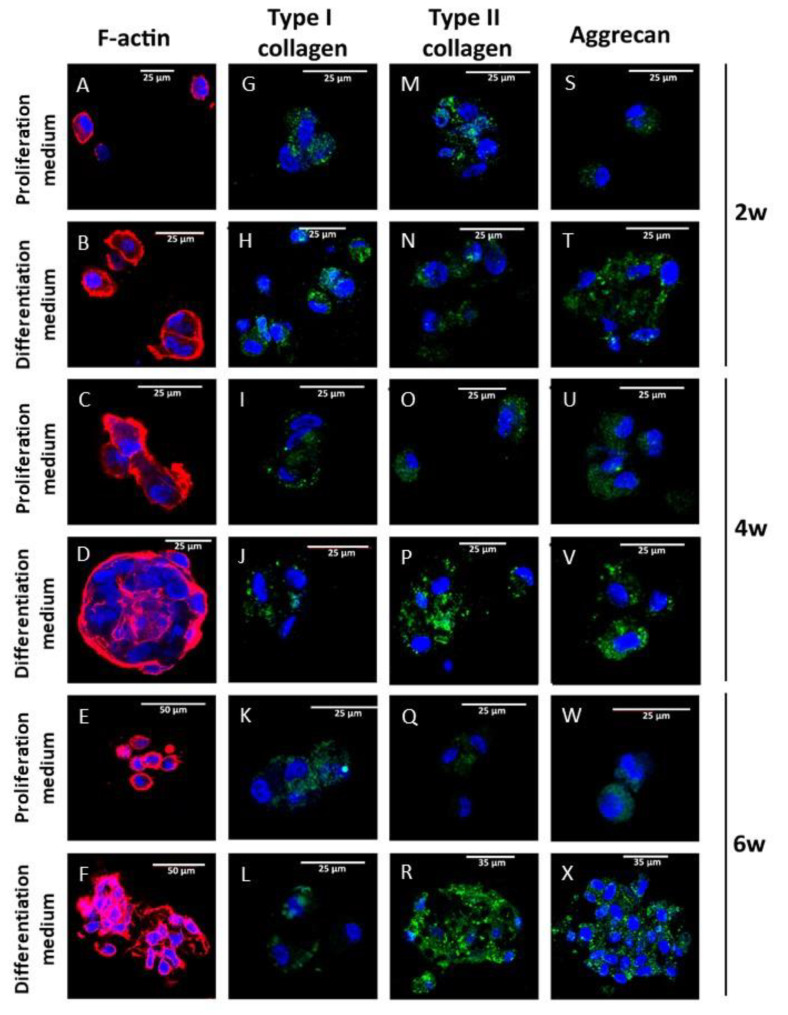
Chondral differentiation in alginate-agarose gels. Human primary chondrocytes were encapsulated in 3% alginate-1% agarose hydrogels and cultured with proliferation or differentiation medium for 2, 4 and 6 weeks at a density of 2 × 10^6^ cells/mL. F-actin organization was evaluated by fluorescence microscopy using rhodamine-phalloidine (**A**–**F**). Type I (**G**–**L**) and type II (**M**–**R**) collagen as well as aggrecan (**S**–**X**) were evaluated by immunofluorescence. Nuclei were stained with DAPI. The images are representative of *n* = 5 different experiments.

The induction of the chondrogenic phenotype in the alginate-agarose scaffolds was analyzed. Cells were cultured in 3% alginate-1% agarose scaffolds for 2, 4 and 6 weeks. Cytoskeletal morphology, distribution and organization were evaluated by F-actin fluorescence staining using rhodamine-phalloidin, as described in Methods. Fluorescence images were obtained by confocal microscopy. The results are summarized in Figure 6A–F. Spheroidal aggregates of cells were observed in all groups analyzed. These aggregates were more abundant and larger in the 4- and 6-week cultures compared to the 2-week groups, in which small groups of 2–4 cells were found. These differences increased when differentiation medium was used, which resulted in larger and more numerous cell aggregates than those observed in cultures with proliferation medium (Figure 6D,F). As observed in the 2D-culture, the F-actin filaments showed a peripheral distribution in the cells. In the cell aggregates, fluorescence staining evidenced peripheral distribution within the aggregates, as observed when serial confocal planes were analyzed (Figure 6D and Supplementary Video S1). 

Type I and II collagen as well as aggrecan were determined by immunofluorescence staining in the same experimental groups. A residual expression of type I collagen was detected in all the experimental groups analyzed, being slightly higher in cells cultured with proliferation medium for 6 weeks (Figure 6K). Type II collagen and aggrecan showed the same trend. The expression of both proteins was higher in cells cultured with differentiation medium for 4 and 6 weeks compared to the corresponding cultures with proliferation medium. Cell aggregates showed a more intense fluorescence staining, concentrated in the central region for both type II collagen and aggrecan, as demonstrated by serial confocal plane images (Figure 6P,V,R,X, and Supplementary Videos S2 and S3). 

### 3.5. Chondral Differentiation of hDPSCs Cultured in Alginate-Agarose Scaffolds

**Figure 7:**
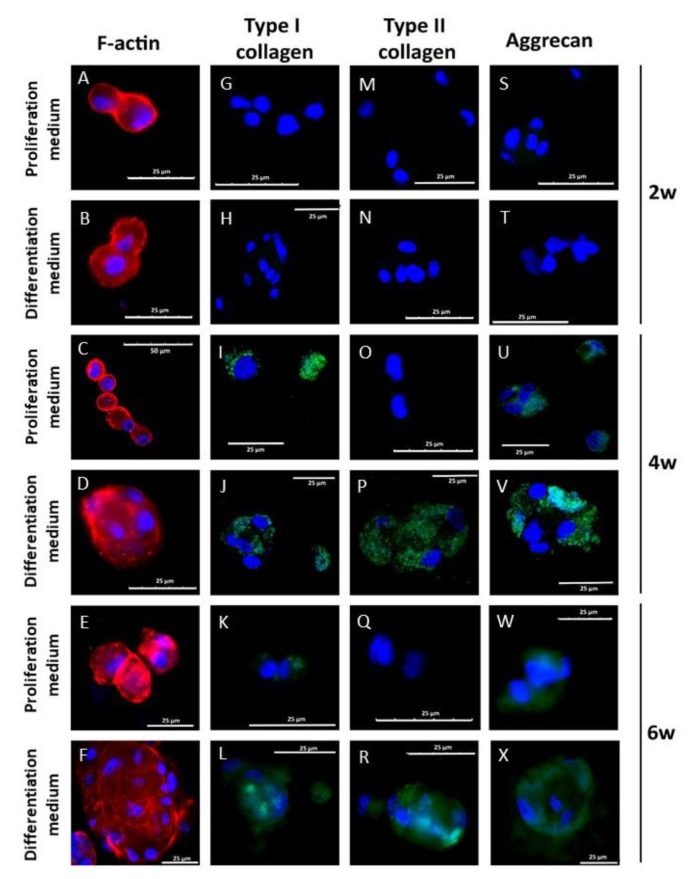
hDPSCs differentiation in alginate agarose gels. 2 × 10^6^ cells/mL were encapsulated in 3% alginate 1% agarose hydrogels and cultured with proliferation or differentiation medium for 2, 4 and 6 weeks. F-actin organization was evaluated by fluorescence microscopy using rhodamine-phalloidine (**A**–**F**). Type I (**G**–**L**) and II (**M**–**R**) collagen as well as aggrecan (**S**–**X**) were evaluated by immunofluorescence. Nuclei were staining with DAPI. Images are representative of *n* = 5 different experiments.

In a similar way as described above, the chondrogenic differentiation of hDPSCs cells cultured in 3% alginate-1% agarose scaffolds was studied. The results obtained are summarized in Figure 7. F-actin staining showed the presence of large cell aggregates in samples cultured with differentiation medium for 4 and 6 weeks. Small aggregates were found in cells cultured with proliferation medium. The cells cultured in the scaffolds acquired a rounded morphology, with few F-actin filaments within the cells (Figure 7A–F). Type I collagen expression was detected in all experimental cultures at week 4 and 6, and no differences were observed between samples cultured with proliferation or differentiation media (Figure 7G–L). However, type II collagen was only detected in cells cultured with differentiation medium for 4 and 6 weeks (Figure 7M–R). Regarding aggrecan expression, it was observed in cells cultured for 4 and 6 weeks in both differentiation and proliferation culture media (Figure 7S–X).

### 3.6. Morphometric Analysis of Cell Aggregates in Alginate-Agarose Scaffolds

A morphometric analysis was performed to estimate the density and size of the cell aggregates found in the alginate-agarose scaffolds, as described in Methods. Both chondrocytes and hDPSCs were cultured in 3% alginate-1% agarose scaffolds with proliferation or differentiation media for 6 weeks. The results obtained are represented in Table 3. With respect to the chondrocyte cultures, the number of aggregates showed a 10-fold significant increase when cultured with differentiation medium compared to those cultured with proliferation medium, although no change was observed in their mean diameter. We also observed a significant 8-fold increase in total cell number in the cultures with differentiation medium compared to those cultured with proliferation medium as well as a significant increase in the number of non-forming-aggregate cells. No differences were observed in the number of cells per aggregate or in the cell diameter. 

In contrast, in hDPSCs cultures, the number of cell aggregates was significantly lower but with a higher diameter in samples cultured with differentiation medium, compared to those cultured with proliferation medium, which were then significantly more abundant but smaller. In the hDPSCs samples cultured with proliferation medium there were significant increase of total number of cells as well as of non-forming-aggregates cells. No differences in the number of cells per aggregate or in cell diameter were found. 

### 3.7. Analysis of the Expression of Chondrogenesis-Related Genes

**Figure 8:**
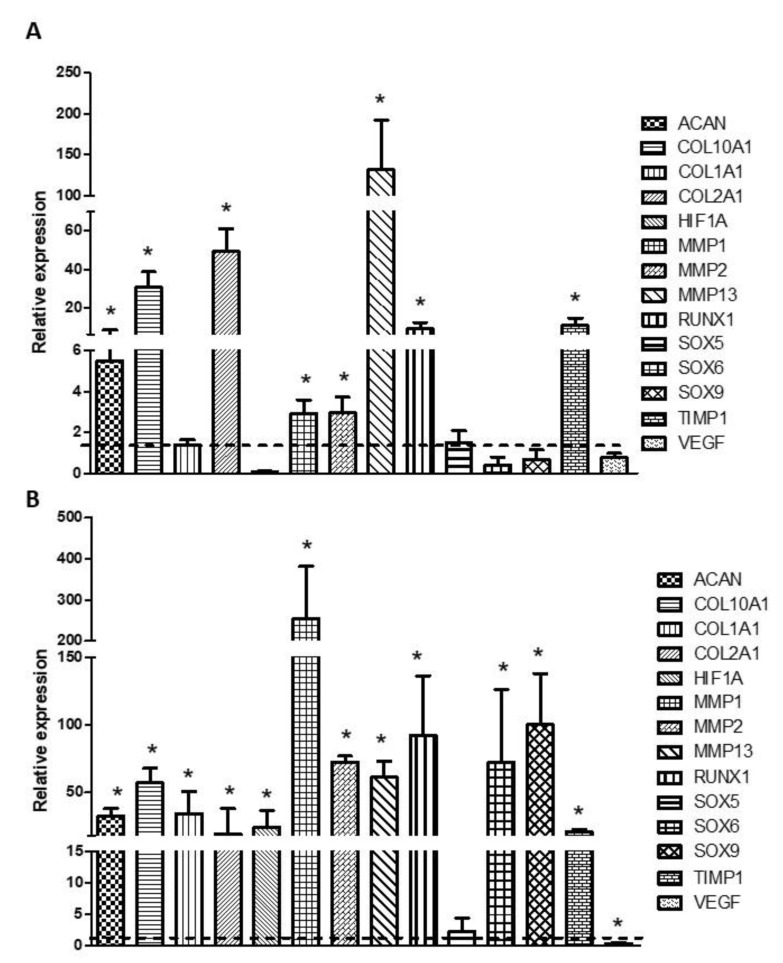
Levels of expression of chondrogenesis-related genes. Human primary chondrocytes (**A**) or hDPSCs (**B**) were encapsulated in 3% alginate-1% agarose hydrogels and cultured with proliferation or differentiation medium for 6 weeks at a density of 2 × 10^6^ cells/mL. Relative levels of gene expression were evaluated by real-time RT-PCR. GAPDH was used as a housekeeping gene. The fold change (2-∆∆Ct) was calculated using the relative expression of cells cultured with proliferation medium as control group (indicated by the dotted line). Mean ± SD of three different experiments is represented. * *p* < 0.05 compared to control group.

Finally, the expression of several genes involved in the chondrogenic differentiation process was studied in chondrocytes and hDPSCs cultured in 3% alginate-1% agarose hydrogels, with proliferation or differentiation media. The results obtained are represented in Figure 8. Both ACAN and COL2A1 are overexpressed in chondrocytes and hDPSCs samples cultured with differentiation medium compared to those cells cultured with proliferation medium. The differentiation medium did not induce the expression of COL1A1 in chondrocytes samples, whereas a significant upregulation was detected in hDPSCs samples. The expression of COL10A1 but not of VEGF was induced in both cell types cultured with differentiation medium. The expression levels of MMP1, 3 and 13, as well as those of TIMP1 and RUNX1 were significantly higher in both cell types cultured with differentiation medium. The expression of SOX6 and 9, as well as HIF1A, were also induced, but only in hDPSCs samples. Finally, no variation in SOX5 expression was detected in any cells type incubated with differentiation medium. 

## 4. Discussion

The primary goal of this study was to optimize an approach based on the MACI technique that would be potentially useful for its application in cartilage tissue engineering. We previously reported preliminary results supporting the use of alginate in combination with both chondrocytes and hDPSCs for cartilage regeneration in vitro and in vivo [21]. Alginate hydrogels have also been used for cartilage regeneration, with the induction of cartilage ECM synthesis and chondrogenesis. Negative charges present in the alginate chemical structure are known to induce the retention of newly synthesized aggrecan molecules. However, the limitations of this material include weak mechanical stability, slow degradation, and poor cell adhesion [34,35,36]. On the other hand, agarose has been extensively used in biomedical applications because of its controlled self-gelling properties, water solubility, adjustable mechanical properties, and non-immunogenic properties. Agarose, based on its stiffness and functional groups, can also support cellular adhesion and proliferation, among other activities. In fact, agarose has an adjustable water adsorption capacity, which provides a suitable microenvironment for cellular activity [23]. It presents excellent mechanical properties, besides being non-immunological, presenting great biocompatibility, absence of toxicity, and high cell/matrix interaction, which has emphasized the application of agarose in the cartilage repair research [25]. Agarose mixed with alginate has been used for cartilage regeneration. Agarose improves biomechanical properties of alginate as well as its chondrogenic properties. The combination of alginate-agarose has been demonstrated to shear thinning properties, and provide a yield strength similar to those of Pluronic, a synthetic polymer composed of PEO–PPO–PEO triblock copolymers of poly(ethylene oxide) (PEO) and poly(propylene oxide) (PPO), commonly used in biomedicine applications. The use of agarose−alginate mixtures has been studied for applications such as bioprinting, showing improved print-shape fidelity when compared to agarose gels only [24,25,26].

For obvious reasons, chondrocytes are the optimal cell type to be used for cartilage regeneration. However, its poor proliferative capacity and its tendency to dedifferentiate, as well as other disadvantages in relation to the extraction source (cartilage biopsy, which is not exempt from morbidity) limit its used for cartilaginous tissue engineering approaches [21,37]. These limitations have motivated researchers to investigate the use of other cell types. Among them, MSCs of different origins, including bone marrow or adipose tissue, have been evaluated because of their ability to regenerate cartilage [37]. In recent years, hDPSCs have become a realistic alternative to be used in MACI approaches due to several factors, including their high proliferative capacity while maintaining the ability to differentiate into multiple lineages, including chondral cells [13,21]. In agreement with other researchers, we found that both chondrocytes and hDPSCs expressed type II collagen and aggrecan in 2D-cultures in the presence of differentiation medium. This expression paralleled the appearance of characteristic morphological changes of differentiated chondrocytes, and were more evident in large cell aggregates, which were specially found in primary chondrocyte cultures, as discussed below. The relative expression of genes related to chondral differentiation was analyzed to complement the morphological results obtained in 2D-cultures. As expected, and according to the immunofluorescence results, COL2A1 and ACAN were up-regulated in both cell types cultured with differentiation medium. Regarding COL1A1, a significant overexpression was found in hDPSCs cultured with differentiation medium compared to cells cultured with proliferation medium. The differentiation medium contains TGF-beta, which induces the expression of procollagen 1 in hDPSCs [38]. Moreover, hDPSCs in culture secrete TGF-beta, which could stimulate hDPSCs in a paracrine manner to expressed COL1A1 [39]. It is important to note that the gene expression data are not presented in an absolute but a semi-comparative way, and that the actual amount of type 1 collagen synthetized is not significant, as evidenced by the results of immunofluorescence staining.

RUNX1 participates in early chondrogenic differentiation, unlike other members of the RUNX family such as RUNX2. RUNX1 has been reported to be widely expressed in chondrocyte progenitor cells and to stimulate chondrogenesis [40,41]. Furthermore, RUNX1 contributes to the production of chondral matrix by enhancing type 2 collagen expression in cooperation with SOX proteins [42]. In accordance with this observation, we found a significant overexpression of both RUNX1 and SOX9 when differentiation medium was used. 

Type X collagen is the main marker for hypertrophic chondrocytes, and it is expressed in the first days of chondrogenic differentiation [43]. Although type X collagen overexpression is associated with cartilage hypertrophy, the role of this protein during cartilage development remains under investigation. Knuth et al. demonstrated the role of type X collagen in chondrogenic differentiation of pellet-cultured MSCs using knockdown experiments. They found that the silencing of COL10A1 resulted in significantly smaller pellets than those obtained from non-knockdown cells, and a significant decrease in the expression of GAGs and ACAN, as well as in the gene and protein expression of type II collagen. Similarly, we found a significant induction of COL10A1 in chondrocytes and hDPSCs cultured with differentiation medium, but the expression profile of the cells analyzed as well as the absence of induction of VEGF, another key regulator of hypertrophy, led us to discard that the cells developed a hypertrophic phenotype.

Next, the chondrogenic capacity of the alginate-agarose hydrogels containing chondrocytes and hDPSCs was evaluated. As expected, agarose improved the biomechanical properties of the alginate scaffolds. We did not observe a substantial loss in the consistency of the scaffolds throughout the culture time, although it would be interesting to study whether the cells cultured in hydrogels modify their rheological properties. Large scaffolds (0.5 mL, 3 cm length) were made because we were interested in designing a useful system for subsequent application in vivo. As the proliferation of chondrocyte limits their use in vivo, a low cell density was used (2 × 10^6^ cells/mL, which is less than half of those used by other authors) [44,45]. This low cell density has been shown to be efficient in in vivo articular cartilage regeneration models using chondrocytes and hDPSCs in an alginate gel [21]. We observed a homogeneous distribution of cells within the scaffolds, demonstrating the excellent diffusion of nutrients and gases through the manufactured hydrogels, supporting the usefulness of manufactured scaffolds to generate large cartilage substitutes for the in vivo treatment of large lesions [27]. Both chondrocytes and hDPSCs cultured in mixed alginate-agarose hydrogels acquired a rounded morphology and as expected, expressed a significant amount of aggrecan and type II collagen, especially when cultured with differentiation medium. This agrees with López-Marcial et al. [5], who demonstrated that chondrocytes cultured in alginate-agarose hydrogels secreted large amounts of GAGs and proposed this mixed hydrogel as effective for the additive manufacture of cartilage substitutes. Residual levels of type I collagen expression were detected in chondrocytes samples as well as basal expression level in hDPSCs samples, especially in those experimental groups cultured with proliferation medium. However, the expression of type II collagen and aggrecan was significant higher in hDPSCs cultured with differentiation medium, which agrees with the chondrogenic differentiation capacity reported for these cells [21]. 

The formation of cell aggregates is a key step in the shaping of cartilage. Many classical studies have not only demonstrated that high cell density is a requirement for chondrogenesis, but have also correlated the extent of cell condensation with the level of chondrogenesis [46]. In chondrocyte aggregates, cell-cell communication mediated by gap junctions stimulates the expression of the chondral matrix components. Furthermore, the formation of these cell aggregates induces the chondrogenic phenotype and the maintenance of cultured chondrocytes [46,47,48,49]. In the same way, cell aggregation induces the differentiation of MSCs into chondrocytes through mechanisms that involve not only the secretion of cell signaling molecules like TGF-beta or N-CAM, but also interactions with the extracellular matrix [46]. Before aggregation, MSCs secrete hyaluronan and type I collagen, but when aggregated, a cellular switch occurs with the secretion of type II collagen in parallel with morphological changes, including the acquisition of a rounded shape [46]. The data provided here is in line with this evidence. hDPSCs, stimulated by the 3D environment and the medium composition, acquired a rounded shape, and expressed large amounts of type II collagen and aggrecan. Type I collagen expression decreased but was still detectable, which could indicate an early state of differentiation of hDPSCs, which is the contrary to what happened with chondrocytes. Due to the importance of aggregate formation, we decided to quantify the number and size of aggregates in our scaffolds. An aggregate was considered when it was formed by at least 4 cells, which is the minimum number to consider a coronary group in native cartilage [1]. As expected, more aggregates were found in the primary chondrocytes samples when cultured with differentiation medium. Regarding the hDPSCs, more aggregates were observed in the scaffolds cultured with proliferation medium, but their average size was significantly smaller than those cultured with differentiation medium. Cell aggregation of MSCs inhibits cell proliferation, which explains our results since the number of total hDPSCs was higher in scaffolds cultured with proliferation medium that in those samples cultured with differentiation medium. We also measured the size of individual cells but found no significant differences between cells cultured with either medium used, which strengthens the absence of a hypertrophic phenotype. A notable issue is that the manufactured scaffolds induce the spontaneous formation of cell aggregates and, as observed in scaffolds containing chondrocytes cultured with proliferation medium, cell aggregation of cells is enough stimulus to induce the chondral phenotype. 

The analysis of the gene expression of COL1A1, COL2A1 and ACAN confirmed the results observed in the immunofluorescence studies. COL1A1 overexpression was found in hDPSCs. As discussed above, this induction could be related to the formation of the aggregates, which is consistent with morphological studies, in which we observed that hDPSCs, but not chondrocytes, formed fewer and smaller aggregates when cultured with differentiation medium. This could be because hDPSCs acquire the chondral phenotype more slowly. In the same line as in 2D cultures, an induction of the gene expression of COL10A1 but not of VEGF was observed, which could be indicative of an early stage in cartilage development rather than an acquisition of a hypertrophic phenotype. Morphometric measurements of cells size in the scaffold support this hypothesis. 

In both chondrocytes and hDPSCs an increase of MMPs expression was observed. Although MMPs are associated with osteoarthritis, it seems that, in fact, it is the balance between MMPs and TIMP that induces degeneration of the articular cartilage [50]. In this way, we also observed an increase in the expression of TIMP1 that, supported by the evidence discussed above, probably indicates a remodeling process rather than an acquisition of a hypertrophic phenotype, which agrees with the overexpression observed of the SOX and RUNX1 genes.

In summary, the results presented here support the efficacy of mixed alginate-agarose scaffolds for cartilage regeneration in vitro. Although chondrocytes have shown better performance than hDPSCs, these stem cells could be used in those situations where it is not possible to obtain autologous chondrocytes. Nevertheless, it is important to highlight that other important characteristic of hDPSCs, such as their anti-inflammatory properties [21], have not been evaluated in this work, so in vivo experiments are necessary to explore them. 

## Figures and Tables

**Table 1:** Genes IDs and corresponding assay numbers of transcripts analyzed by real-time RT-PCR.

Gene ID	Gene Name	Assay Number	2D or 3D Cultures
ACAN	Aggrecan	Hs00153936_m1	2D and 3D
COL10A1	Collagen Type X Alpha 1 Chain	Hs00166657_m1	2D and 3D
COL1A1	Collagen Type I Alpha 1 Chain	Hs00164004_m1	2D and 3D
COL2A1	Collagen Type II Alpha 1 Chain	Hs00264051_m1	2D and 3D
GAPDH	Glyceraldehyde-3-Phosphate Dehydrogenase	4325793	2D and 3D
HIF1A	Hypoxia Inducible Factor 1 Subunit Alpha	Hs00153153_m1	3D
MMP1	Matrix Metallopeptidase 1	Hs00899658_m1	3D
MMP13	Matrix Metallopeptidase 13	Hs00233992_m1	3D
MMP2	Matrix Metallopeptidase 2	Hs01548727_m1	3D
RUNX1	RUNX Family Transcription Factor 1	Hs01021971_m1	3D
SOX5	SRY-Box Transcription Factor 5	Hs01552796_m1	3D
SOX6	SRY-Box Transcription Factor 6	Hs00264525_m1	3D
SOX9	SRY-Box Transcription Factor 9	Hs00165814_m1	2D and 3D
VEGFA	Vascular Endothelial Growth Factor A	Hs00900055_m1	2D and 3D

**Table 2:** Mechanical testing of alginate-agarose hydrogels for a torque of 50 µN*m.

[Alginate] (%)	[Agarose] (%)	G’ (Pa)	G’’ (Pa)	E (Pa)
1.5	0	NA	NA	NA
3	0	NA	NA	NA
0	0.5	718.09 ± 199.03	52.41 ± 13.74	2160.01
0	1	1856.02 ± 212.39	123.31 ± 7.36	5580.34
1.5	1	1177.78 ± 104.23	72.08 ± 14.49	3539.95
3	1	1247.27 ± 210.81	101.78 ± 27.27	3754.26

NA: not applicable.

**Table 3:** Morphometric analysis of cell aggregates in alginate-agarose scaffolds.

	Chondrocytes		hDPSCs	
	PM	DM	PM	DM
**N** **umber of aggregates (*n*)**	8 ± 0.7	93 ± 11.2 (*p* = 0.009) *	73 ± 0.6	38 ± 0.2 (*p* = 0.0009) *
**Number of cells per aggregate (*n*)**	4.9 ± 4.9	6.9 ± 1.4 (*p* = 0.219)	4.0 ± 0.4	6.8 ± 3.1 (*p* = 0.108)
**Maximum** **Ø of aggregates (** **µm)**	42.5 ± 30.4	55.8 ± 8.8 *(p* = 0.205)	29.2 ± 2.5	50.2 ± 16.9 (*p* = 0.044) *
**Number** **of non-aggregate-forming cells (n)**	61 ± 8.2	154 ±17.9 (*p* = 0.033) *	247 ± 22	93 ± 8.2 (*p* = 0.0001) *
**Total number of cells (n)**	107 ± 11	817 ± 76 (*p* = 0.007) *	547 ± 37	287 ± 21 (*p* = 0.0003) *
**Cell** **Ø (** **µm)**	33.6 ± 4.8	32.4 ± 3.4 (*p* = 0.544)	24.4 ± 1.9	27.6 ± 7.2 (*p* = 0.152)

PM: proliferation medium; DM: differentiation medium. Mean ± SD of three different experiments is represented. * *p* < 0.05 compared to PM group.

## Data Availability

All the data generated in this research are included in the manuscript.

## Supplementary Materials

The following are available online at https://www.mdpi.com/article/10.3390/biomedicines9070834/s1, Video S1: Acting fluorescence staining of a cell aggregate. Video S2: Type II collagen immunofluorescence staining of a cell aggregate. Video S3: ACAN immunofluorescence staining of a cell aggregate.
